# Persistent primitive lateral basilovertebral anastomosis-rethinking vertebrobasilar junction fenestration

**DOI:** 10.1007/s00276-025-03714-9

**Published:** 2025-09-03

**Authors:** George Triantafyllou, Panagiotis Papadopoulos-Manolarakis, Nikolaos-Achilleas Arkoudis, George Velonakis, Alexandros Samolis, Maria Piagkou

**Affiliations:** 1 Department of Anatomy, School of Medicine, Faculty of Health Sciences, National and Kapodistrian University of Athens, 75 Mikras Asias Str., Goudi, 11527 Athens, Greece; 2 “VARIANTIS” Research Laboratory, Department of Clinical Anatomy, Mazovian Academy in Plock, Plock, Poland; 3 Department of Neurosurgery, General Hospital of Nikaia-Piraeus, Athens, Greece; 4 Research Unit of Radiology and Medical Imaging, National and Kapodistrian University of Athens, Athens, Greece; 5 Second Department of Radiology, General University Hospital “Attikon”, National and Kapodistrian University of Athens, Athens, Greece

**Keywords:** Primitive lateral basilovertebral anastomosis, Vertebrobasilar junction, Fenestration, Arterial ring, Computed tomography angiography, Neuroradiological anatomy

## Abstract

**Purpose:**

To describe and analyze two rare cases of arterial rings at the vertebrobasilar junction (VBJ), likely representing persistent segments of the primitive lateral basilovertebral anastomosis (PLBVA), and to explore their embryological origin and clinical significance.

**Materials and methods:**

Two morphological arterial variants were identified during a retrospective review of computed tomography angiography (CTA) scans from 505 patients. Multiplanar reconstruction and three-dimensional volume rendering were used for anatomical characterization.

**Results:**

Two cases (0.4%) showed arterial rings at the VBJ, formed by a variant vessel arising from a vertebral artery and reconnecting with the basilar artery shortly after its origin. The vascular arrangements aligned with persistent segments of the PLBVA rather than true fenestrations. No significant asymmetry, hypoplasia, or dominance was seen in the vertebral or basilar arteries.

**Conclusion:**

VBJ arterial rings may be embryological remnants of the PLBVA, a rare and often overlooked vascular variation. While usually asymptomatic, their presence can complicate radiologic interpretation and endovascular procedures. Proper identification through detailed CTA assessment is critical to avoid diagnostic errors and ensure procedural safety.

## Introduction

The cerebral arterial circle, or Circle of Willis, is formed by the terminal branches of the internal carotid artery (ICA) and the vertebrobasilar system (VBS). Posterior circulation is supplied by the bilateral vertebral arteries (VAs), which join to form the basilar artery (BA) before splitting into the posterior cerebral arteries [11]. Although this configuration is often described as anatomically consistent, various morphological variants, especially within the vertebrobasilar junction (VBJ), have been documented through both cadaveric and imaging studies.

Among these, arterial fenestrations and rings in the posterior circulation are relatively uncommon findings. In our previous meta-analysis, we estimated the prevalence of intracranial VA fenestration to be approximately 0.3% [12]. In contrast, the most extensive 3D-CTA study of 16,416 patients reported a 1.29% prevalence for BA fenestrations [10]. Importantly, such configurations can mimic or obscure vascular pathologies during neuroradiological assessments or endovascular procedures [14].

Beyond these anatomical duplications, another category of vascular variation involves the persistence of embryonic arteries. The most common is the persistent trigeminal artery (PTA), a remnant of early carotid–basilar anastomosis [13]. Less frequently, other transient fetal vessels may persist, including the olfactory artery and the primitive lateral basilovertebral anastomosis (PLBVA), a longitudinal embryologic vessel hypothesized to contribute to some VBJ arterial rings [3, 9].

In a retrospective review of CTA scans of the posterior circulation, we identified two vascular ring formations involving the VBJ. These configurations do not conform to the classic definitions of fenestration or duplication and may represent persistent embryological anastomoses rather than postnatal vascular remodeling. This paper aims to describe these cases, examine their morphological features, and discuss their embryological origin and potential clinical relevance.

## Anatomic variation

As part of a retrospective angiographic investigation, two computed tomography angiography (CTA) scans were identified from an archived imaging database for their unique vertebrobasilar morphology. The dataset was sourced from the General Hospital of Nikaia–Piraeus, Greece, under the auspices of ethical approval (*Protocol No. 56485; Approval Date: November 13, 2024*). Image review and post-processing were conducted using Horos software version 3.3.6 (Horos Project). Anatomical assessments were based on axial, coronal, and sagittal multiplanar reconstructions (MPR), as well as three-dimensional volume-rendered reconstructions.

### Case #1

**Fig. 1:**
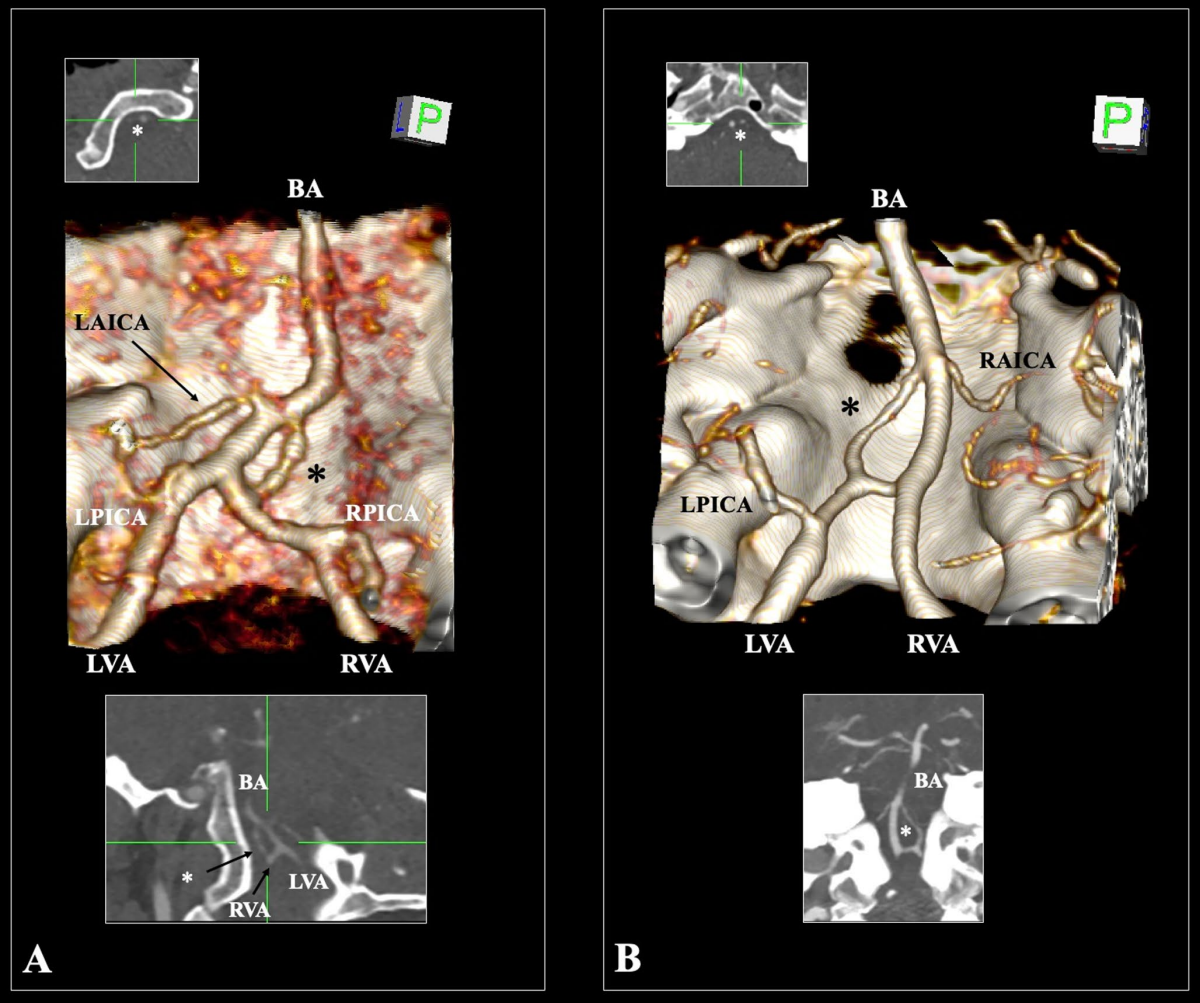
**A** Three-dimensional volume-rendered and multiplanar reconstructed (MPR) computed tomography angiography (CTA) images demonstrating a rare arterial ring at the vertebrobasilar junction (VBJ) in Case #1. The left and right vertebral arteries (LVA and RVA) join to form the basilar artery (BA). A variant arterial branch (marked with asterisks- *) arises from the RVA and reconnects with the BA approximately 3.4 mm distal to its origin, forming a complete arterial ring. The posterior inferior cerebellar arteries (PICA) originate normally from the V4 segments of both VAs. The left anterior inferior cerebellar artery (LAICA) is also visible coursing laterally. **B** CTA images of Case #2 showing an arterial ring configuration at the VBJ. The LVA and RVA merge to form the BA. A variant arterial branch (marked with an asterisk- *) originates from the LVA and reanastomoses with the BA distal to its origin, forming a distinct vascular ring. The left PICA (LPICA) and right anterior inferior cerebellar artery (RAICA) originate from their typical locations. MPR reconstructions confirm the complete continuity of the ring

The first case involved a 53-year-old male patient who underwent CTA for evaluation of the posterior circulation. The left vertebral artery (LVA) had a diameter of 2.6 mm, while the right vertebral artery (RVA) measured 2.9 mm, indicating near-symmetrical dominance. The posterior inferior cerebellar arteries (PICA) originated bilaterally from the V4 segment of the vertebral arteries. The BA measured 2.6 mm in diameter and 30.2 mm in length. Approximately 3.4 mm distal to the VBJ, an atypical arterial branch was identified, originating from the RVA and coursing medially to reconnect with the BA. This vessel measured 7.0 mm in length and 1.8 mm in diameter, forming a complete arterial ring in conjunction with the BA. The ring measured 7.4 mm in longitudinal length and 2.6 mm in maximal transverse width, as confirmed by 3D and MPR imaging (Fig. 1A). No arterial wall irregularities, stenoses, or significant dominance were noted in the VBS. The vascular configuration is consistent with a persistent segment of the PLBVA, rather than a classical fenestration, given the branch’s independent origin and distinct reconnection pathway (Gailloud et al. 2002) [3].

### Case #2

The second case concerned an 81-year-old female patient who also underwent CTA of the posterior circulation. The LVA measured 3.4 mm in diameter, while the RVA was 2.9 mm, indicating mild left-sided dominance. The two VAs fused to form the BA, which had a diameter of 3.0 mm and a total length of 31.3 mm. Approximately 3.2 mm distal to the VBJ, a variant arterial branch was visualized arising from the LVA and coursing toward the BA, terminating in a secondary anastomotic connection. This vessel measured 12.7 mm in length and 1.9 mm in diameter. It formed an arterial ring with the BA, measuring 10.1 mm in length and 5.6 mm in width (Fig. 1B). The configuration was confirmed through MPR and VR reconstructions. The trajectory and morphology of this vessel strongly suggest a persistent segment of the PLBVA—an embryonic longitudinal anastomosis that typically regresses during VB development. These findings align with the embryological descriptions by Gregg and Gailloud [3] and Rusu et al. (2024), further differentiating the ring from a true BA fenestration.

## Discussion

The two unusual morphologies forming arterial rings at the VBJ in our report have a strong embryological background. The VBS develops from a complex process involving the longitudinal neural arteries, cervical intersegmental arteries, and several temporary embryonic connections [7, 13]. Bonasia et al. [1] emphasized that the longitudinal neural arteries are initially discontinuous channels, later unified by transverse anastomoses, which explains why fenestrations and duplications can persist when this fusion process is incomplete. Initially, the posterior circulation is supplied by presegmental arteries originating from the primitive ICA, which connect to the hindbrain and spinal vessels. The VAs form through the longitudinal anastomosis of the first six cervical intersegmental arteries. Meanwhile, the BA develops through the cranial fusion of the longitudinal neural arteries and caudal input from the pro atlantal artery’s radiculomedullary branches [4]. A key embryonic structure in this process is the PLBVA, a lateral channel connecting the vertebral system with cerebellar and brainstem arteries. Bonasia et al. [1] noted that failure of regression of such primitive channels can yield ring-like vascular morphologies, often indistinguishable from “true” fenestrations unless their embryological origin is considered. However, its partial persistence has been associated with adult vascular variants such as cerebellar artery anomalies, arterial rings, and VB duplications [3, 9]. Therefore, fenestrations and duplications of the VBS arise from two embryonic mechanisms: (1) failure of longitudinal neural arteries that leads to the classical description of fenestrations, or (2) persistence of embryonic arteries, such as PLBVA and proatlantal remnats, which lead to anomalous channels and rings—similar to the current cases [1].

In our cases, the ring formations observed at the VBJ likely represent persistent segments of the PLBVA, as supported by their anatomical trajectory and separate origins, which are inconsistent with classical fenestrations. This supports the theory that some morphological variants occur due to delayed regression or stabilization of primitive embryonic channels.

In a CTA-based analysis of 3327 patients, Uchino et al. [14] reported 93 fenestrations, with only 6 cases (0.18%) located at the VBJ. A closer inspection of the illustrated cases suggests that some may depict persistent PLBVA segments forming arterial rings. In our retrospective review of 505 cases, two VBJ rings were identified, indicating a prevalence of 0.4%, which is higher than previously recognized and warrants further investigation.

Several case studies support these interpretations. De Caro et al. [2] were the first to describe PLBVA in cadaveric dissection. Their case differed due to vessel hypoplasia and marked vertebral dominance. Yagi et al. [5] identified a ruptured aneurysm associated with a PLBVA during digital subtraction angiography (DSA), emphasizing its clinical relevance. Gregg & Gailloud [3] provided embryological illustrations of PLBVA persistence resembling our observations. Rusu et al. [9] described PLBVA variants coexisting with other anomalies, including cerebellar artery duplications and irregularities in the circle of Willis.

Clinically, while PLBVA-related rings are often asymptomatic and incidental, their relevance becomes evident in several contexts. First, these persistent vessels may influence cerebral hemodynamics, creating foci for aneurysm formation or vascular turbulence [6]. Second, their presence complicates surgical planning, diagnostic interpretation, and endovascular navigation. Misidentifying a PLBVA as a pathological fenestration, duplication, or aneurysmal sac could lead to diagnostic and therapeutic errors [8]. Lastly, Pedro et al. [8] demonstrated that such variants may compromise collateral flow and stroke compensation, especially in posterior circulation ischemia.

This study presents certain limitations inherent to its retrospective, observational design. First, the sample size, although moderate at 505 CTA scans, limits broader generalizability and may underrepresent the true prevalence of rare vascular variants, such as the PLBVA. Second, the absence of correlative DSA or histopathologic validation restricts the definitive classification of vascular rings as persistent embryonic structures rather than postnatal morphological remodeling. Third, while 3DVR and MPR provide detailed anatomical insight, dynamic hemodynamic assessment (e.g., using computational flow modeling or perfusion studies) was not performed and could further elucidate the physiological relevance of such variants. Finally, the study design does not allow conclusions regarding the potential clinical outcomes of these morphological variants. Future prospective studies incorporating functional imaging and longitudinal follow-up may better clarify the role of persistent PLBVA in cerebrovascular pathophysiology.

In conclusion, vertebrobasilar rings caused by persistent PLBVA segments are a rare but important anatomical variation. Recognizing them, using multiplanar and 3D CTA reconstructions, is crucial for safe surgical and diagnostic procedures. A better understanding of embryological vascular remnants may enhance the detection and interpretation of posterior circulation anomalies.

## Data Availability

No datasets were generated or analysed during the current study.
