# Correction: Persistent primitive lateral basilovertebral anastomosis-rethinking vertebrobasilar junction fenestration

**DOI:** 10.1007/s00276-025-03787-6

**Published:** 2025-12-02

**Authors:** George Triantafyllou, Panagiotis Papadopoulos-Manolarakis, Nikolaos-Achilleas Arkoudis, George Velonakis, Alexandros Samolis, Maria Piagkou

**Affiliations:** 1 Department of Anatomy, School of Medicine, Faculty of Health Sciences, National and Kapodistrian University of Athens, 75 Mikras Asias Str., Goudi, 11527 Athens, Greece; 2 VARIANTIS” Research Laboratory, Department of Clinical Anatomy, Mazovian Academy in Plock, Plock, Poland; 3 Department of Neurosurgery, General Hospital of Nikaia- Piraeus, Athens, Greece; 4 Research Unit of Radiology and Medical Imaging, National and Kapodistrian University of Athens, Athens, Greece; 5 Second Department of Radiology, General University Hospital “Attikon”, National and Kapodistrian University of Athens, Athens, Greece

**Correction to: Surgical and Radiologic Anatomy (2025) 47:197** 10.1007/s00276-025-03714-9

The article “Persistent primitive lateral basilovertebral anastomosis-rethinking vertebrobasilar junction fenestration”, written by George Triantafyllou, Panagiotis Papadopoulos-Manolarakis, Nikolaos-Achilleas Arkoudis, George Velonakis, Alexandros Samolis, and Maria Piagkou was originally published electronically on the publisher’s internet portal on 3rd September 2025 without open access. With the author(s)’ decision to opt for Open Choice the copyright of the article changed to © The Author(s) 2025 and the article is forthwith distributed under a Creative Commons Attribution 4.0 International License, which permits use, sharing, adaptation, distribu tion and reproduction in any medium or format, as long as you give appropriate credit to the original author(s) and the source, provide a link to the Creative Commons licence, and indicate if changes were made. The images or other third party material in this article are included in the article’s Creative Commons licence, unless indicated otherwise in a credit line to the material. If material is not included in the article’s Creative Commons licence and your intended use is not permitted by statutory regulation or exceeds the per mitted use, you will need to obtain permission directly from the copyright.

The original article has been corrected.

